# Implementation of a Mentoring Program for Mentee-Mentor Satisfaction: A Longitudinal Pilot Study

**DOI:** 10.1007/s40670-024-01994-1

**Published:** 2024-02-09

**Authors:** Michael C. David, Melissa A. Pitman

**Affiliations:** 1 School of Medicine and Dentistry, Griffith University, Gold Coast, QLD Australia; 2 The Daffodil Centre, The University of Sydney, a Joint Venture With Cancer Council, Sydney, NSW 2011 Australia; 3 St Paul’s College, Sydney, NSW Australia

**Keywords:** **L**ongitudinal, Medicine, Mentoring, Mentor, Mentee, Quantitative

## Abstract

**Introduction:**

Mentoring is a unique educational workplace relationship that can support both the mentee and mentor’s skill, knowledge, social, and emotional needs. The primary aim of this longitudinal pilot study was to implement a formal mentoring program to assess its effect on mentee and mentor satisfaction.

**Methods:**

Data was collected from two hospitals in New South Wales, Australia in late 2018 and early 2019. Junior doctors (mentees) and senior medical staff (mentors) were asked to complete pre-, mid-, and oost-program surveys, with questions relevant to mentee-mentor satisfaction, interactions, and participation. Mixed-effects ordinal logistic regression models were used to assess the program effect on mentee-mentor satisfaction, while Fishers’ exact test was used to evaluate mentee-mentor interactions and participation.

**Results:**

Although there was evidence of upwards trends in the proportion of mentees and mentors who reported their satisfaction in the program as excellent and rated their work satisfaction as being very influenced by the program, both trends were statistically non-significant. While our study was likely underpowered, high participation rates provide promising evidence of the program’s acceptability and feasibility.

**Conclusion:**

Though not reaching statistical significance, study results suggest that the implementation of a mentoring program has the potential to increase satisfaction levels among its participants, be they mentees or mentors. It is recommended that future studies recruit larger samples thereby having sufficient statistical power. Furthermore, causality should be explored in more detail through a multi-site randomized controlled trial design.

## Introduction

Industry and academia have frequently used mentoring to support and build professional relationships [1–3]. Mentoring has been identified as a valuable tool for junior doctors’ personal and professional development [4, 5]. Mentoring is considered distinct from all formal assessment processes within this setting, including educational and clinical supervision, although some individuals may play both supervisor and mentor roles. For example, the Royal College of Physicians Core Medical Training curriculum in the UK currently includes the objective of “being willing to accept mentoring as a positive contribution to promote personal professional development” [6]. It is vitally important to differentiate the role of a mentor from that of an advisor, supervisor, or another supportive senior colleague. However, mentoring often occurs in advising or supervisory relationships, and there usually is a continuum regarding the extent to which such relationships represent mentoring [7]. Compared to other supportive working relationships, the mentoring relationship is characterized by more shared intent and higher levels of involvement by both parties [8, 9]. It is more transformational than transactional.

Though medical schools are increasingly incorporating mentorship into their programs [10, 11], there remains a paucity of publications describing or evaluating mentorship programs in medical education research concerning the degree of program satisfaction [12, 13]. Over the past 30 years, research into the beneficial effects of mentoring has predominately been confined to the business sector [14, 15]. Fortunately, and as Hansford et al. [16] reported in a systematic review, such benefits are generalizable to other fields, such as education and medicine. Furthermore, these benefits are not seen to be one-dimensional. The research literature clarifies that benefits emanating from mentoring programs are multi-dimensional, encompassing both mentor and mentee [11, 17].

Firstly, there are numerous benefits to serving as a mentor compared to those who have never had the opportunity to mentor. Mentors typically are more productive, have greater career satisfaction, and report more personal gratification [18, 19]. They also have enhanced competence, greater access to new ideas, and experience more personal growth [20]. Further, in some settings, they gain increased recognition from their peers [18]. Secondly, mentoring can benefit the institution where it is implemented [18]. This happens through greater job satisfaction, more capable staff, higher levels of motivation and productivity, and the creation of lifelong learning norms. Thirdly, there are the benefits that mentoring programs bestow on mentees. Mentees are more likely to develop competencies, improve their performance, and have greater professional and personal satisfaction in a medical setting than those without access to a mentoring program [21]. Those who receive quality mentoring have been shown to accrue more objective benefits later in their career paths, such as better compensation, more publications and grants, a greater likelihood of being promoted, more recognition, and enhanced career mobility and opportunity [22]. In addition, in terms of their subjective experience, they are more likely to report greater role socialization, have more satisfaction with and commitment to their careers, express more positive beliefs and confidence about their capacity to advance professionally, feel more prepared to be leaders, and believe that the mentoring experience supported their personal development [23].

Mentoring programs are increasingly being implemented based on this theoretical background. However, the extent to which a mentoring program can support specific learning needs, especially in the medical sector, has rarely been explored using a longitudinal framework [24]. This study addresses this situation by analyzing participant’s (program mentees and mentors) satisfaction with a hospital mentoring program for junior doctors in the Central Coast Local Health District (CCLHD) of New South Wales, Australia.

Therefore, the primary aim of this study was to undertake an exploratory analysis of the program’s effect on satisfaction among CCLHD medical personnel, be they senior or junior medical staff, firstly with the program itself and, secondly, concerning their careers. A secondary aim was to assess the acceptability and feasibility of the program using participation as a marker of these implementation measures.

## Methods

### Participants

Participants in the voluntary mentoring program were recruited from 61 junior doctors in training and 50 senior medical staff practicing at two hospitals within CCLHD: (1) the 484-bed Gosford District Hospital (GDH) and (2) the 300-bed Wyong Public Hospital (WPH). Located between Sydney and the Hunter Valley, the CCLHD provides health services to over 330,000 people in the Gosford City and Wyong Shire local government areas [25].

### Mentoring Program

Training and information regarding the mentoring program were delivered over several sessions. Commencing in late 2018 and concluding in early 2019, mentors predominately attended these meetings. The first of these sessions was the *Introductory Mentor Training* session. Potential mentors at GDH and WPH were provided with information and resources relating to mentoring in general and the mentoring program. There were 25 attendees at the GDH session, which was held in November 2018 and 10 at the WPH session, which was held in December 2018. Subsequently, a *Mentor/Mentee Meet and Greet* session was held in January 2019. At this informal session, mentors and mentees were matched based on information about career goals and developmental concerns to establish mentor–mentee relationships outside the confines and restrictions of work and training. It was held 2 months before the first of three surveys. In June 2019, 1 month before the second survey was released, another session with mentors was conducted. Titled *Developing Mentor Skills*, it provided a forum for mentor feedback and fostered the development of mentoring skills through role-plays in several different scenarios. This session had 15 attendees. Finally, a *Mentoring Program Wrap-up* session was held in December 2019, 2 months before the third and last survey. Attendees included both mentees and mentors and numbered 35. The type of mentee-mentor meeting was optional, with all meeting forms allowable, be they face-to-face, virtual, email, telephone, or others.

### Data Collection

Participants were surveyed three times. Each of these surveys was by way of an anonymous, voluntary, electronic survey via email to all potential participants. The email contained a link to an electronic survey engine, *Survey Monkey* (Survey Monkey, Palo Alto, California), where participants could complete the survey and provide feedback. Participants were asked to give the last four digits of their mobile phone number as an identifier for the longitudinal analysis.

The first survey, the pre-program survey, was released on the 11th of March 2019. The second survey, the mid-program survey, was released on the 18th of July 2019, and the third and final survey, the post-program survey, was released on the 15th of February 2020. Each survey remained available for four weeks, with the pre-program survey focusing on matters such as resources and mentee-mentor allocation.

### Outcome Measures

Across the three surveys, the number of items ranged from seven to 18 for mentees and nine to 17 for mentors. Except for age, all items were measured on a binary or ordinal scale. Items aligned with the study’s primary aim were the questions “Overall, please indicate your satisfaction with the program so far” (excellent vs. satisfactory vs. needs improvement) and “Has the current mentoring program beneficially influenced your satisfaction in working at CCLHD?” (very influenced vs. moderately influenced vs. somewhat influenced vs. indifferent vs. not influenced). The number of surveys completed, type of mentee-mentor interaction, and number of mentee-mentor meetings were used to investigate the secondary aim of this study.

### Ethical Considerations

The CCLHD Operational Research Committee approved this study as being of “Negligible ethical risk,” as it only involved the use of non-identifiable data, and applied no more risk to any patient than inconvenience. It did not present any other potential ethical risks to those involved. Participation was based on informed consent.

### Statistical Analysis

Descriptive statistics were examined and reported for continuous data as medians and interquartile ranges (IQR) due to non-normality. Categorical data were reported as counts and percentages. Between-group comparisons of continuous variables were performed using the Mann–Whitney *U* test, while categorical variables were compared using Fisher’s exact test. In addition, mixed-effects ordinal logistic regression modeling was used to assess the temporal effect of the mentoring program on the two satisfaction outcome measures. All reported *p*-values were based on two-sided tests and compared to a significance level of 0.05. A complete case analysis was performed, and missing data were not imputed. Analyzes were performed with Stata Version 17.0 (StataCorp, College Station, Texas).

## Results

### Participant Characteristics

Participant characteristics at the pre-program stage are reported in Table 1. Forty (93%) of the 43 eligible participants completed the pre-program survey. As expected, mentors were found to be significantly older than mentees (*p* < 0.01), with median ages being 53 years (IQR: 47–59) and 27 years (25–29), respectively. A significant gender difference (*p* = 0.04) was observed, with mentees more likely to be female (53% vs. 32%), while mentors were more likely to be male (63% vs. 16%). Nine participants (22%) did not specify their gender. While mentors (38% vs. 11%) were more likely to have had prior experience with a mentoring program, this difference was not statistically significant (*p* = 0.07).

**Table 1:** Characteristics of mentoring program participants (mentees = 19; mentors = 21)^a^ at the pre-program stage

**Participant characteristic**	**Total**	**Mentees**	**Mentors**	***p*-value**
	***N* (%)**	***N* (%)**	***N* (%)**	
**Age (years), Median (IQR)**	34 (27–53)	27 (25–29)	53 (47–59)	< 0.01^b^
**Gender**				0.04^c^
Female	13 (33)	10 (53)	3 (16)	
Male	18 (45)	6 (32)	12 (63)	
Missing	9 (22)	3 (15)	6 (21)	
**Prior experience (as a mentee and/or mentor) with a formal mentoring program**				0.07^c^
No	30 (75)	17 (90)	13 (62)	
Yes	10 (25)	2 (11)	8 (38)	
Missing	0 (0)	0 (0)	**0 (0)**	

*IQR* interquartile range

^a^Three eligible participants did not complete the pre-program survey

^b^Mann-Whitney *U* test

^c^Fisher’s exact test

### Participation Rates, Types of Interactions, and Number of Meetings

Of the 22 mentees and 21 mentors enrolled in the program, no significant difference (*p* = 0.71) in participation rates between the two groups (*p* = 0.71) was detected, with 14 (64%) mentees and 16 (76%) mentors completing all three surveys (see Table 2); the resulting overall response rate for all surveys was 70%. Non-response was more likely on the post-program survey, with six mentees (27%) and five mentors (24%) failing to respond, compared to less than 10% for the pre-and mid-program surveys, irrespective of the participant being a mentee or mentor.

**Table 2:** Interaction, meeting and survey^a^ characteristics of mentees (*N* = 22) and mentors (*N* = 21)

	**Mentees *n* (%)**^**b**^	**Mentors *n* (%)**^**b**^	***p*-value**^**c**^
**Surveys completed**			
0	0 (0)	0 (0)	0.71
1	3 (14)	1 (5)	
2	5 (23)	4 (19)	
3	14 (64)	16 (67)	
**Type of primary interaction**			
** Primary interaction**			0.43
Face-to-face	13 (59)	16 (76)	
Email	2 (9)	0 (0)	
Telephone	1 (5)	0 (0)	
Others	0 (0)	0 (0)	
Missing	6 (27)	5 (24)	
** Secondary interaction**			0.12
Face-to-face	3 (14)	0 (0)	
Email	6 (27)	7 (33)	
Telephone	7 (32)	5 (24)	
Others	0 (0)	4 (19)	
Missing	6 (27)	5 (24)	
**Initiator of meetings**			
** Mid-program**			0.15
Participant	10 (45)	6 (29)	
Equally responsible	8 (36)	6 (29)	
The other participant	2 (9)	8 (38)	
Missing	2 (9)	1 (5)	
** Post-program**			0.31
Participant	8 (36)	3 (14)	
Equally responsible	7 (32)	10 (48)	
The other participant	1 (5)	3 (14)	
Missing	6 (27)	6 (29)	
**Number of meetings**			
0–2	4 (18)	2 (10)	0.52
** 3**	4 (18)	8 (38)	
4+	8 (36)	6 (29)	
Missing	6 (27)	5 (24)	

^a^Surveys conducted pre-, mid- and post-mentoring program

^b^Due to round-off, percentages do not always sum to 100%approach

^c^Fisher’s exact test

In their post-program surveys, program participants were asked, “What was the primary method used for interaction?” and “What was the secondary method used for interaction?” Recollections from both groups were very similar (see Table 2), with no evidence of a significant difference in the former (*p* = 0.43) or latter (*p* = 0.12). Face-to-face meetings as the primary interaction were substantially more popular (mentees = 59%; mentors = 76%) than other types of interaction such as email, telephone, or other. This was not the case with secondary interactions, with mentees slightly preferring telephone calls (32%) to email (27%) and the converse for mentors.

Participants were also asked in their mid- and post-program surveys, “Who predominately made contact to arrange meetings?” While not statistically significant, Table 2 shows mentees during mid-program (45% vs. 29%) and post-program (36% vs. 14%) were more likely than mentors to initiate interactions.

Participants were also asked on the post-program survey, “How many times did you meet your mentee/mentor throughout the program?” (see Table 2). Of those who responded, mentees were more likely to participate in at least four meetings (*n* = 8, 36%), while mentors were more likely to participate in three meetings (*n* = 8, 38%). No significant difference was detected between the two groups (*p* = 0.52).

### Program Satisfaction and Influence

As reported in Table 3, the proportion of mentees rating the program as providing excellent satisfaction increased from 21% (*n* = 5) pre-program to 50% (*n* = 11) post-program, while the proportion among mentors increased only slightly from 24% (*n* = 5) to 28% (*n* = 6). However, mixed-effects ordinal logistic modeling to assess the program’s effect over time did not detect any significant change in program satisfaction for either mentees (*p* = 0.42) or mentors (*p* = 0.27). Similarly, while the proportion of mentees and mentors rating the program as very influenced career satisfaction increased from mid-program (mentees: 5%; mentors: 5%) to post-program (mentees: 18%; mentors: 10%), overall, mixed-effects ordinal logistic modeling indicated that the program effect for both mentees and mentors was not statistically significant (mentees: *p* = 0.06; mentors: *p* = 0.16).

**Table 3:** Program satisfaction among mentees (*N* = 22) and mentors (*N* = 21)

	**Pre-program *N* (%)**	**Mid-program *N* (%)**	**Post-program *N* (%)**	***p*-value**^**a**^
**Satisfaction with program**				
**Mentees**				0.42
Excellent	5 (21)	10 (45)	11 (50)	
Satisfactory	13 (59)	8 (36)	3 (14)	
Needs improvement	1 (5)	2 (9)	2 (9)	
Missing	3 (14)	2 (9)	6 (27)	
**Mentors**				0.27
Excellent	5 (24)	3 (14)	6 (28)	
Satisfactory	15 (71)	14 (67)	10 (48)	
Needs improvement	1 (5)	3 (14)	0 (0)	
Missing	0 (0)	1 (5)	5 (24)	
**Influence of program on work satisfaction**				
**Mentees**				0.06
Very influenced	Not applicable^b^	1 (5)	4 (18)	
Moderately influenced	Not applicable^b^	10 (45)	9 (41)	
Somewhat influenced	Not applicable^b^	7 (32)	2 (9)	
Indifferent	Not applicable^b^	0 (0)	1 (5)	
Not influenced	Not applicable^b^	2 (9)	0 (0)	
Missing	Not applicable^b^	2 (9)	6 (27)	
**Mentors**				
Very influenced	Not applicable^b^	1 (5)	2 (10)	0.16
Moderately influenced	Not applicable^b^	3 (14)	4 (19)	
Somewhat influenced	Not applicable^b^	6 (29)	4 (19)	
Indifferent	Not applicable^b^	3 (14)	3 (14)	
Not influenced	Not applicable^b^	7 (33)	3 (14)	
Missing	Not applicable^b^	1 (5)	5 (24)	

^a^Mixed-effects ordinal logistic regression with an unstructured covariance, with time (pre-program, mid-program, post-program) being the only independent variable

^b^‘Influence of program on work satisfaction’ not included in the pre-program survey

## Discussion

Though significance was not reached, the proportions of participants reporting high level of satisfaction with the program and its influence on their careers at CCLHD at post-program was found to be, at the very least, similar to those at pre-program. In addition, proportions of participants reporting a degree of non-satisfaction (needs improvement) and/or lack of influence (indifferent or not influenced) were seen to be less at post-program compared to mid-program. These results reflect past findings that mentoring is not mutually exclusive and is more likely to benefit both parties involved in the mentoring process—not just the mentee [23, 26–29].

In addition, the overall proportion of those who completed all three surveys (30/43 = 70%) was higher than the pooled rate from a recent systematic review by Van Gelder et al. [30], which pooled results from 169 observational studies, thereby suggesting a more than an adequate degree of study acceptability and feasibility among participants. Face-to-face meetings were found to be the most popular type of interaction, similar to findings recently reported by Raghunanda et al. [31]. As a second preference, email and telephone communications were of similar frequencies.

The main strengths of this study were that it was both longitudinal and quantitative, unlike the majority of studies that have focused on mentoring programs, which in the main have been cross-sectional and/or qualitative [10, 32–34]. Secondly, but most importantly, mentors were allowed to receive training and resources before the program commenced. A lack of appropriate training can lead to the delivery of inappropriate advice or the imposition of the mentor’s views on the mentee, resulting in conflict and disillusion [28, 35].

There are several caveats to our findings. Firstly, surveys were done via an online questionnaire, thus generating concerns regarding potential selection and reporting biases [36, 37]. That being said, the response rate was relatively high for both mentors and mentees at all time points. Secondly, there was no control group to confirm the effects of the mentoring program. This study cannot ascribe change in outcome measures to the program without a control. Further investigation would need to include a control to assess causality more directly. Thirdly, and due to the small sample size, it may be well that the study was underpowered, thus producing type II errors and non-significant test results. Fourthly, estimation of program effect could have been enhanced if more survey items had allowed ordinal or continuous scale responses rather than dichotomous responses. It has been shown that reliance on the latter leads to a loss of information and power [38], as the latter are more likely to produce biased estimates due to social desirability bias [39]. Lastly, all participants were recruited from two hospitals within the CCLHD, one of 15 local health districts in NSW, which, as a consequence, might limit our findings’ generalizability. To rectify this, in addition to being randomized, it is recommended that future studies be expanded to include hospitals from the other 14 local health districts.

Although the impact and importance of mentorship have been discussed at length, tangible suggestions for improving mentoring programs are equally valuable, especially with the purpose of extending this program beyond CCLD. Firstly, it would also be beneficial if eligible non-participants were identified, such as junior doctors deemed to be in most need of a mentee-mentor relationship, plus mentors who are genuinely vested in the mentoring process, and having high concordance with the interests and goals of mentees [40]. Secondly, gender imbalance in mentor participation needs to be addressed. While mentors of either gender should be equally effective, especially for female members, to support their academic activities [41–43], female mentors might be necessary for female mentees for reasons, such as their ability to serve as role models in combining the demands of their career with family commitments and better understand female mentees undergoing training [42, 44, 45]. Therefore, it is necessary to cultivate and recruit female senior medical staff as mentors.

## Conclusion

This pilot study addressed a gap in current research on this topic. While not reaching statistical significance, the results suggest that the implementation of a mentoring program has the potential to increase career satisfaction among its participants. Consequently, it is recommended that future studies recruit large samples and use multi-site randomized controlled designs, thereby having increased statistical power to determine the causal effect of the program’s effect on satisfaction among medical personnel.

## Data Availability

The raw data collected for the current study are available from the corresponding author on reasonable request.

## Code Availability

Code is available from the corresponding author on reasonable request.
